# Hydrogels for Single-Cell Microgel Production: Recent Advances and Applications

**DOI:** 10.3389/fbioe.2022.891461

**Published:** 2022-06-17

**Authors:** B. M. Tiemeijer, J. Tel

**Affiliations:** ^1^ Laboratory of Immunoengineering, Department of Biomedical Engineering, TU Eindhoven, Eindhoven, Netherlands; ^2^ Institute of Complex Molecular Systems, TU Eindhoven, Eindhoven, Netherlands

**Keywords:** single cell, hydrogel, droplet, microfluidics, microgel, immunology, pairing

## Abstract

Single-cell techniques have become more and more incorporated in cell biological research over the past decades. Various approaches have been proposed to isolate, culture, sort, and analyze individual cells to understand cellular heterogeneity, which is at the foundation of every systematic cellular response in the human body. Microfluidics is undoubtedly the most suitable method of manipulating cells, due to its small scale, high degree of control, and gentle nature toward vulnerable cells. More specifically, the technique of microfluidic droplet production has proven to provide reproducible single-cell encapsulation with high throughput. Various in-droplet applications have been explored, ranging from immunoassays, cytotoxicity assays, and single-cell sequencing. All rely on the theoretically unlimited throughput that can be achieved and the monodispersity of each individual droplet. To make these platforms more suitable for adherent cells or to maintain spatial control after de-emulsification, hydrogels can be included during droplet production to obtain “microgels.” Over the past years, a multitude of research has focused on the possibilities these can provide. Also, as the technique matures, it is becoming clear that it will result in advantages over conventional droplet approaches. In this review, we provide a comprehensive overview on how various types of hydrogels can be incorporated into different droplet-based approaches and provide novel and more robust analytic and screening applications. We will further focus on a wide range of recently published applications for microgels and how these can be applied in cell biological research at the single- to multicell scale.

## Introduction

The vast complexity of the human body is gradually being unraveled. However, the more we discover about various types and sub-populations of cells, the more questions are often raised. Furthermore, the complex web of intercellular interactions they maintain makes fully understanding the depth of biological processes and regulators challenging. Over the years, research has downsized by moving from a tissue, to cell population, and to single-cell resolution, in order to grasp the most basic interactions underlying systemic responses (Altschuler and Wu, 2010; Satija and Shalek, 2014). This miniaturization has not only allowed the reduction of noise in measuring systems but also allowed more precise measurement, smaller sample sizes, and less reagent consumption. In addition, it has broadened the view on how homogenous, cellular behavior within well-defined populations of cells, really is. As technology advanced and cell behavior was studied at a smaller and smaller level, it became clear that cell populations displayed much more heterogenous behavior than previously thought (Woodland and Dutton, 2003a; Gordon and Taylor, 2005; Villani et al., 2017). This allowed for characterization of vast amounts of sub-populations of cells with specific specializations. By looking at individual cells instead of populations, the masking cloud of averaged measurements could be elevated, showing that some cells are more potent at specific tasks than others (Dueck et al., 2016). This way of looking at cellular heterogeneity could explain the fact that cell-therapies are often less effective *in vivo* as they are predicted to be *in vitro,* and often vary immensely between subjects (Chattopadhyay et al., 2014; Satija and Shalek, 2014). To fully dissect the complexity of cellular heterogeneity and cell–cell interactions, reliable methods of high-throughput single-cell research must be developed. The developments in the field of microsystems and microfluidics have proven to be a valuable tool in establishing such novel approaches.

Since the rise of soft lithography (Xia and Whitesides, 1998), microfluidics has been making major leaps forward with increasingly complex device designs (Murphy et al., 2017; Shinde et al., 2018; Jammes and Maerkl, 2020). Generally, these have consisted of intriguing labyrinths of channels connected to low-volume fluidic pumps. These designs allow for very low sample sizes but high precision experimental setups, which efficiently scales down but improves experimental control. More importantly, the micrometer scale is ideal for physical manipulation of cells using fluidics (Sims and Allbritton, 2007; Shinde et al., 2018; Luo et al., 2019; Yeh and Hsu, 2019). The relatively gentle nature of moving cells around with fluids puts it at a high advantage over mechanical approaches. Nevertheless, the production of these conventional microfluidic devices with various capture chambers, wells, and often multilayered channels can be tedious and generally the throughput is limited by the device dimensions.

Droplet-based microfluidics uses relatively simple device designs in which laminar flow under low Reynolds number allows for fast, reliable, and reproducible droplet production (Shang et al., 2017). These droplets, ranging from sizes in the pico-to nanoliter scale can be used as a tool to encapsulate and thus spatially control cells, similar to what capture chambers and wells aim to do in conventional microfluidic devices. In droplets however, the number of processed cells is only limited by the available amount of reagent, as samples are continuously flushed through the microfluidic device. This potential for theoretically unlimited throughput together with flexibility and a huge range of applications has allowed droplet-based microfluidics to rapidly become a discipline of its own (Shembekar et al., 2016; Sinha et al., 2018).

Even though variations exist, typical droplet-based approaches for cell encapsulation utilize two phases of fluids. The dispersed phase is a water-based cell suspension, and the continuous phase is an oil. This approach has gained much interest as the focus of cell biological research has shifted from studying cell populations to conducting experiments with single-cell resolution (Matuła et al., 2020). Droplet microfluidics has proven suitable to encapsulate these single cells, bringing them from a “bulk” cell suspension to a “single-cell” suspension where cells are separated by water–oil interfaces. However, capturing cells in oil also makes it very difficult to further influence, manipulate, measure, or process them without breaking the emulsion and returning to the “bulk” cell suspension (Luo et al., 2019).

**FIGURE 1:**
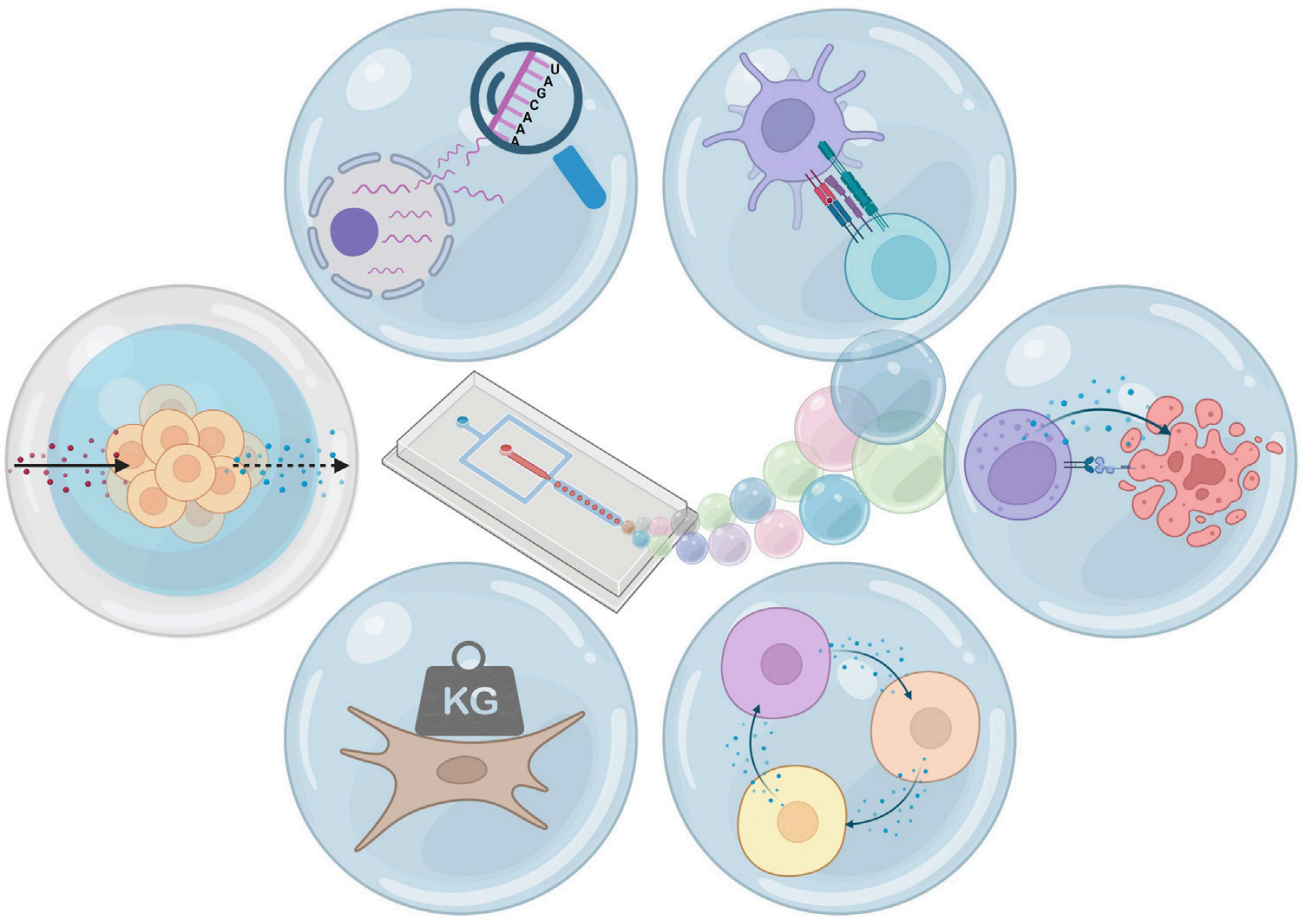
Hydrogel microfluidic droplets: applications. From top-left clockwise: single-cell sequencing, pairing for cell interaction, pairing for cytotoxicity, coculture, changing and measurement of mechanical properties, and selectively permeable hydrogel shells. Figure created using Biorender.

Over recent years, the combination of hydrogel and microfluidic droplets has been applied to maintain spatial control over cells after de-emulsification or to provide cells with a solid droplet environment (Zhu and Yang, 2017; Kamperman et al., 2018; Mohamed et al., 2020). Various hydrogels, gelation methods, droplet production techniques, and cell types gave rise to large amounts of unique research (Goy et al., 2019). This strong combination is utilized for a variety of innovative single cell techniques, which can be applied to *in vitro* analytical approaches (Figure 1). Here, we will discuss the current state-of-the-art of single- and multicell droplet techniques, and how these can be complemented with the use of hydrogel. We will start with discussing the basic principles of microfluidics and droplet formation and how commonly used hydrogels can be integrated into this approach. We will then discuss various microgel applications, starting with a single-cell analysis and single-cell pairing and how these can benefit from microgels, followed by the application of semi-permeable hydrogel shells, and finishing with microgel coculture. We believe recent advances in this field demonstrate that this multifaceted combination will allow for promising new applications, facilitate a high-throughput droplets analysis, or provide more suitable culture conditions for adherent cells.

## Hydrogel Droplets

### Microfluidics

**FIGURE 2:**
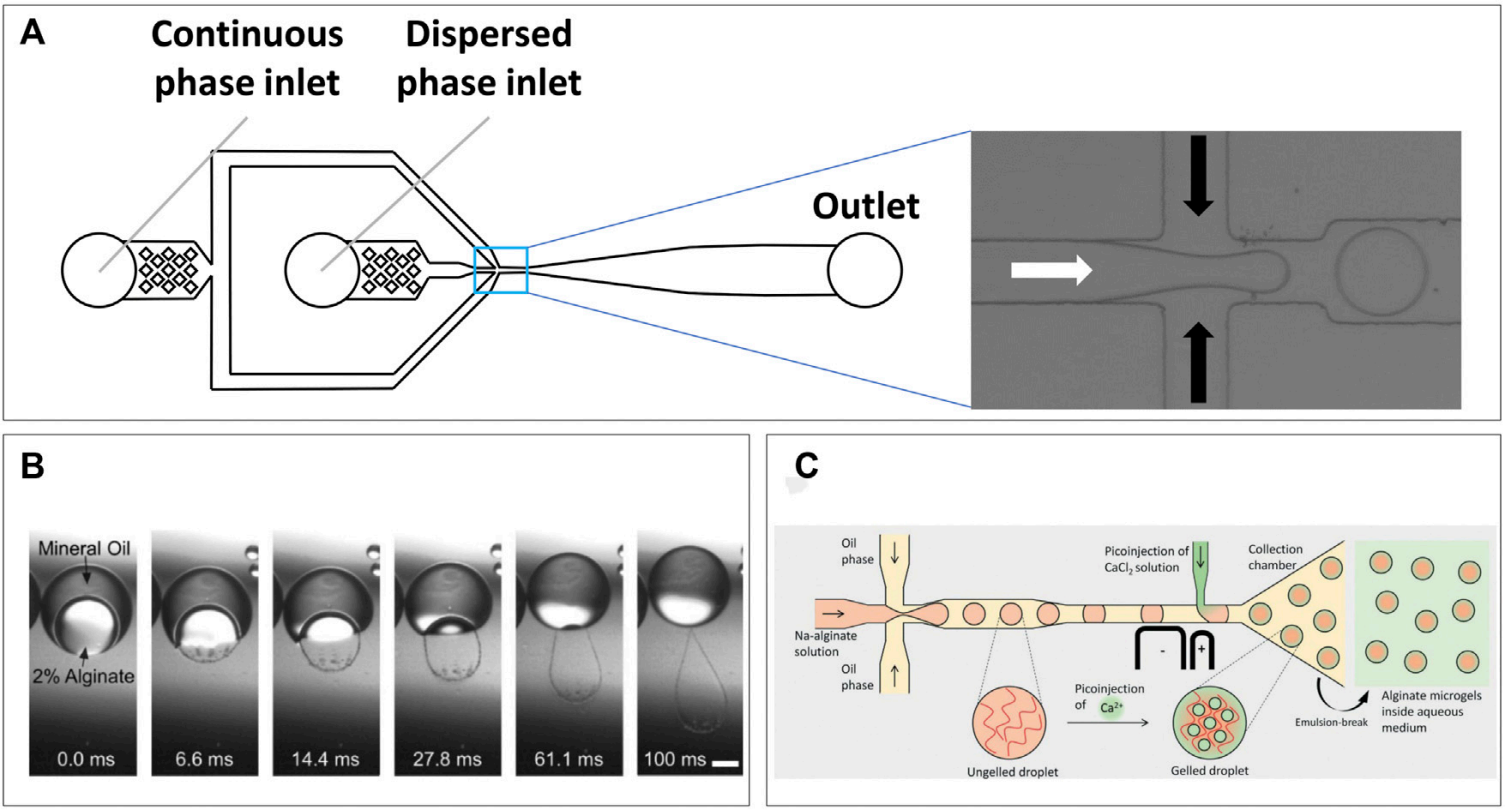
Microfluidic droplet formation. **(A)** Generic emulsification device, two inlets for the continuous and dispersed phases which mix at the channel intersection (pop-out), droplets then continue flowing toward collection from outlet. **(B)** Transfer of alginate solution from a double-emulsion toward a CaCl_2_ solution causes gelation within 100 ms (Martinez et al., 2012). **(C)** Microfluidic design utilizing pico-injection of CaCl_2_ after droplet formation point to prevent premature gelation of alginate solution (Ahmed et al., 2021).

Microfluidic devices for droplet production are commonly produced using soft lithography of polydimethylsiloxane (PDMS) (Xia and Whitesides, 1998). The technique has persisted and remained relevant over years of research due to its ease of use, low cost, and precision, which together allow for fast prototyping of novel microfluidic designs. Such prototyping has mostly been an advantage for complex microfluidic systems, whereas for droplet production, the basics of device designs have remained mostly the same. A generic droplet device consists of two inlets for the continuous and the dispersed phase followed by an outlet for collecting the produced emulsion (Figure 2A). Inside the device, co-flow, flow-focusing, or T-junction geometries ensure the controlled mixing of the two immiscible fluids, which due to laminar flow produces highly homogenous droplet sizes. The distribution of cells in the dispersed phase follows a Poisson distribution (Collins et al., 2015), allowing droplets to be tuned to contain multiple cells or approach single-cell encapsulation. The concepts behind the fluid dynamics of droplet formation have been extensively described previously (Cubaud and Mason, 2008; Shembekar et al., 2016; Tawfik, Griffiths; Zhu and Wang, 2017), and will therefore not be discussed in detail.

When droplet volume and cell concentration are tuned correctly, this can result in a cell distribution, which closely approaches single-cell encapsulation. Deviation from this optimized point will result in either more empty droplets, or more multicell droplets, as in compliance with Poisson distribution (Collins et al., 2015). Single-cell research is commonly used to deprive cells from cell–cell interactions and discover their innate capabilities and potential for responding to specific stimuli. When comparing droplet-based approaches to well- or trap-based applications, the main advantage is the potential for high-throughput, which is indispensable when screening for rare cell behavior or sub-populations. In addition, the oil–water interface ensures complete isolation between cells, while in some well/trap-based microfluidic approaches paracrine signaling cannot be ruled out (Guldevall et al., 2016; Zhou et al., 2020). However, the absence of cellular adhesion and the difficulty to manipulate cells when in oil–water emulsion are inherent challenges of single-cell droplet encapsulation. The addition of hydrogel in droplets to produce microgels proves a double-edged sword, providing solutions to both problems. Cellular adherence and mechanical cues can be provided *via* biocompatible hydrogels providing a semi-solid extracellular matrix-like environment (Dumbleton et al., 2016; Hasturk and Kaplan, 2019; Tiemeijer et al., 2021). On the other hand, the hydrogel can maintain spatial control over cells after de-emulsification while allowing for diffusion and downstream processing using analytes or other reagents (Leonaviciene et al., 2020; di Girolamo et al., 2020; Yanakieva et al., 2020; Chokkalingam et al., 2013).

Some variations of the basic droplet production approach are needed when hydrogel is added to the dispersed water phase. The implementation strongly depends on the type of hydrogel and method of cross-linking. Droplet breakoff is dictated by the ratio of viscosity between the continuous and dispersed phases (Günther and Jensen, 2006). Thus, premature hydrogel gelation, resulting in increased viscosity of the dispersed phase, is detrimental for a consistent droplet size. Therefore, the type of crosslinking dictates droplet formation and adjustments that need to be made to device design. Commonly used types are ionic cross-linking (Choi et al., 2007; Workman et al., 2007; Utech et al., 2015; Ahmed and Stokke, 2021), photo-cross-linking (Zhao et al., 2016; Mohamed et al., 2019; Nan et al., 2019), and thermo-responsive cross-linking (Dolega et al., 2015; Yanakieva et al., 2020; Tiemeijer et al., 2021; Zhang et al., 2021). By design, the latter two do not require on-chip mixing of different aqueous phases and can still be used in conventional device designs. However, the first might require gelators to be mixed in, which means an extra dispersed phase inlet is required for on-chip mixing, just prior to or right after, droplet formation (figure of various designs). Various types of hydrogels have been used in droplet microfluidics, which can roughly be divided into natural polymers and synthetic polymers (Table 1).

**TABLE 1:** List of recently published research using various types of natural and synthetic hydrogels for innovative single-cell microgel techniques.

Type	Hydrogel	Cross linking	Application	Reference
Natural	Alginate	Calcium release from EDTA complex	Controlled gelation of microgels for cell encapsulation	Utech et al. (2015)
		On-chip picoinjection of CaCl_2_	Picoinjection of CaCl_2_ for controlled gelation of microgels for cell encapsulation	Ahmed and Stokke, (2021)
		On-chip mixing of alginate with Ca chelators	Testing of different chelators for cell encapsulation in microgels	Shao et al. (2020)
		Competitive ligand exchange crosslinking	Cell encapsulation with increased gelation control and improved cell viability	Håti et al. (2016)
		Alginate + CaCl_2_ in oil phase	Monodisperse microgels for cell aggregate encapsulation	Wang et al. (2021)
	Agarose	Cooling below 18°C	Pairing of secreting cell with reporter cell and flow cytometric sorting	Yanakieva et al. (2020)
			Pairing of secreting cell with detection beads for flow cytometric measurement	Chokkalingam et al. (2013)
			Encapsulation of cells to monitor cell egress	Neubauer et al. (2019)
	Gelatin based	Cooling on ice and genipin addition	Monitoring single tumor-cell response to adherence on gelatin microgels	Nan et al. (2019)
		Off-chip UV exposure (GelMA)	Rapid generation of injectable stem cell-laden microgels fore tissue engineering	Zhao et al. (2016)
		On-Chip UV exposure (GelMA)	On-chip gelation and retrieval from oil phase for cell encapsulation	Mohamed et al. (2019)
			On-chip gelation and sorting into aqueous medium	Hong et al. (2012)
	Collagen based	Emulsification in the 37°C oil phase	On-chip gelation for cell encapsulation	Bavli et al. (2021)
		Heating to 37°C (Matrigel^®^)	Production of large monodisperse organoids for drug testing	Zhang et al. (2021)
			Production of endothelial cell organoids	Dolega et al. (2015)
Synthetic	PEG based	Mixing acrylated PEG with dextran	Production of semi-permeable shell capsules for multistep processing of large biomolecules	Leonaviciene et al. (2020)
		Mixing thiolated PEG with PEG-dimaleimide	Production of hydrogel beads with force-responsive fluorescence	Allazetta et al. (2017)
		Mixing vinylsulfone-PEG with thiol-PEG	Production of hydrogel beads with tunable stiffness and tunable RGD functionalization	Rahimian et al. (2019)
		Mixing maleimide-PEG with dithiotrietol	Production of semi-permeable hydrogel shells for immunoassays	Zhu, (2010)
		Mixing of PEGDT with MALDEX	Single-cell encapsulation for culture of clones and subsequent mRNA sequencing	Zhao et al. (2021)
		Mixing of thiol-hyaluronic acid with PEGDA	Monodisperse microgels for cell encapsulation with semi-permeable silica coating	Mazutis et al. (2013)
	Polyisocyanide	Heating to above 15°C	Prolonged encapsulation of adherent cells for culture with cell retrieval afterward due to thermoreversibility	Tiemeijer et al. (2021)

### Hydrogels and Cross Linking

#### Natural Hydrogels

Natural polymers have the advantage of being natively biocompatible and are generally cross-linked *via* relatively mild gelation processes increasing cell survival. They form structures that are very comparable to mammalian extracellular matrix and thus ideal for harboring cells (Gasperini et al., 2014).

Alginate is potentially the most used natural polymer to produce microfluidic hydrogel droplets with. The brown algae–derived polysaccharide has been favored due to its biocompatibility (Lee and Mooney, 2012) and various strategies for cross-linking. The polymer forms a hydrogel either due to lowering of pH or in the presence of divalent cations due to ionic interactions (Gurikov and Smirnova, 2018). These ionic interactions allow the alginate fibers to form a supramolecular “Egg-box” structure (Braccini and Pérez, 2001). In droplet applications, the latter is by far more commonly used and often calcium is used for this as released in a CaCl_2_ solution. This will result in extremely fast gelation, and although it has been demonstrated that droplets can be produced by on-chip mixing (Choi et al., 2007), this will generally result in instant and uncontrolled gelation. Illustrative for this instant gelation was the formation of “rain-droplet” shaped hydrogels when alginate was retrieved from double-emulsions into a CaCl_2_ solution (Figure 2B) (Martinez et al., 2012). Therefore, other strategies can be needed for on-chip droplet formation to ensure constant low viscosity during droplet production. Recently, Ahmed et al. demonstrated a unique device design which produced alginate droplets following a conventional approach but used on-chip pico-injection of CaCl_2_ solution to prevent problems with premature gelation and obtain monodisperse hydrogel droplets (Figure 2C) (Ahmed and Stokke, 2021). Alternatives to CaCl_2_ are partially soluble or insoluble calcium salts such as calcium sulfate (Kong et al., 2003) and calcium carbonate (Tan and Takeuchi, 2007; Workman et al., 2007), respectively. As these have lower solubility in water compared to calcium chloride, gelation occurs slower, although this still proves challenging to control (Kuo and Ma, 2001). A promising approach comes in the triggered release of Ca ions from strong chelators such as EDTA. Calcium–chelator complexes are mixed with alginate solutions where the high chelator affinity prevents direct gelation. By decreasing pH, calcium is released which allows cross-linking. This approach proved suitable to maintain stability of droplet formation while triggering gelation directly on-chip (Shao et al., 2020), or at a later time point by acidifying the continuous oil phase off-chip (Utech et al., 2015). Adaptations of this approach utilize competitive chelator kinetics (Bassett et al., 2016), aiming to have more control over gelation dynamics and improved cell viability (Håti et al., 2016). Alginate has a large pore-size of 5–150 nm (Martinsen et al., 1989), which is dictated by the calcium concentration used for cross linking. Although small molecules can diffuse in, large protein diffusion can be limited (Tanaka et al., 1984), which is most likely due to a combination of a non-homogenous pore size on gelation surface, and protein charge at neutral pH (Smidsrød and Skjåk-Bræk, 1990). This should be considered when designing cell studies in alginate droplets. Although perfectly biocompatible, alginate does not provide cells with adherence and will, thus, have to be functionalized with, for example, Arg-Gly-Asp (RGD) motifs (Yu et al., 2009), or be used in a composite with adherent polymers (Xu et al., 2007; Dixon et al., 2014).

Another commonly used natural polymer is agarose, which is like alginate derived from specific types of algae. Unlike alginate it cross-links due to temperature changes and exhibits hysteresis (Indovina et al., 1979). The polysaccharide dissolves at temperatures around 90°C and cross-links due to hydrogen bonds when cooled to around 35–50°C depending on which type of algae was the source. The polymer chains form helical fibers which aggregate into a 3D supramolecular structure (Xiong et al., 2005). Although these transition temperatures are not suitable for cell applications, agarose can be methylated to lower gelling temperature (Gu et al., 2017). Therefore, ultralow gelling point agarose has proven very suitable for hydrogel droplet encapsulation of cells (Chokkalingam et al., 2013; Sinha et al., 2019; Yanakieva et al., 2020). With a gelling temperature of 8°–17°C, it can be dissolved at high temperatures but used to encapsulate cells safely at much lower biological compatible temperatures, without premature gelation. Gelation can easily be triggered by cooling down anywhere between droplet production and droplet-demulsification. Agarose droplets are especially useful for cell processing after de-emulsification due to their stability and relatively large pore-size, which is partially dependent on polymer concentration (Narayanan et al., 2006). Their pore size within the range of 100–600 nm facilitates diffusion of virtually any soluble molecule including antibodies (∼10 nm (Reth, 2013)), which allows inflow of nutrients or fluorescent markers for analysis. This property combined with their stability at lower temperatures has facilitated a whole-droplet flow cytometric analysis (Chokkalingam et al., 2013), and even sorting (Fang et al., 2017; Yanakieva et al., 2020). Agarose is inherently non-adherent to cells, and thus needs functionalization with extracellular matrix molecules to obtain cellular attachment in droplets (Karoubi et al., 2009). Furthermore, agarose is non-degradable by mammalian cells, and thus bacteria-derived agarose (Fu and Kim, 2010), or production of degradable composite hydrogels (Zhang et al., 2012), is needed to retrieve cells. This can be a limiting factor when downstream cell recovery is required as reheating to above the melting point will kill cells and destroy proteins of interest.

Although alginate and agarose are frequently used and offer several desirable characteristics, they are not inherently cell-adherent. Therefore, an alternative can be connective tissue–derived hydrogels which contain peptide motifs facilitating cell attachment. Collagen (Antoine et al., 2014) and its derivative gelatin (Jaipan et al., 2017) are arguably the most commonly used. Gelatin is obtained by breaking collagen down to single-strain molecules (Kuijpers et al., 1999). Both yield thermo-reversible hydrogels with transition temperatures within biocompatible ranges. Uniquely, collagen will cross-link when heated to physiological temperature (Xu et al., 2009), whereas gelatin will cross-link when it is cooled below 35°C (Hellio and Djabourov, 2006). As collagen is the main component of extracellular matrix, these hydrogels are highly biomimetic and often used in tissue engineering approach due to their biodegradability (Antoine et al., 2014; Tondera et al., 2016). In droplet microfluidics, Matrigel^®^ is a commonly used commercially available collagen-based hydrogel (Dolega et al., 2015; Zhang et al., 2021). Below room temperature allows droplet formation, and by simply bringing droplets to culturing temperature, cross-linking occurs. This process is fully reversible, allowing for cell retrieval. As gelatin-based hydrogels will return to the liquid phase at culture temperatures, these have routinely been chemically modified. A commercially available gelatin-based hydrogel is GelMA, which is enriched with methacrylate groups to facilitate UV-triggered cross-linking (van den Bulcke et al., 2000; Sun et al., 2018). These have allowed for both on-chip (Mohamed et al., 2019; Nan et al., 2019) and off-chip (Zhao et al., 2016) gelation and production of microfluidic droplets. A major downside of collagen and gelatin-based hydrogel droplets is their tendency to merge and aggregate after de-emulsification, as was observed for both Matrigel^®^ and GelMA. This makes downstream processing after removal of the continuous phase very challenging.

#### Synthetic Hydrogels

Examples of synthetic polymers used in production of hydrogel droplet microfluidics are poly(ethylene glycol) (PEG), poly(acrylic acid), poly(vinyl alcohol) (PVA), poly(acrylamide), and their derivatives (Wan, 2012). The main advantages of synthetic over natural polymers are their reproducibility of synthesis and the possibility for chemical modification (Hern and Hubbell, 1998). They are inherently non-adhesive to cells and unless modified they are non-biodegradable. Functionalization with molecules such as Arg-Gly-Asp (RGD) motifs (Sionkowska, 2011; Kim et al., 2019), or mixing of natural and synthetic polymers (Krutkramelis et al., 2016), is therefore required for many cellular applications. Alterations of these polymers can be used to provide useful cross-linking approaches such as photopolymerization as demonstrated in PEG (Young et al., 2013; Xia et al., 2017) and PVA (Zhao et al., 2010). Similarly, combinations of natural and synthetic polymers can be combined with temperature responsive moieties to obtain hydrogels with unique swelling and gelling properties (Jiang et al., 2019a). Such composite hydrogels can also allow for fine-tuning of hydrogel degradation (Benavente Babace et al., 2019; Neubauer et al., 2019), which offers lots of potential for *in vivo* applications. In addition, alterations can facilitate complex techniques that would be extremely difficult or impossible with natural hydrogels. Some examples are detection of cell forces (Allazetta et al., 2017), tuning hydrogel stiffness (Rahimian et al., 2019), and controlling diffusion (Zhu, 2010; Leonaviciene et al., 2020). The possibilities for adaptations to synthetic hydrogels are too wide to be covered in this review, but its applications for tissue engineering purposes are very adequately discussed in a review by Jumnin Zhu (White et al., 2021).

### Recovery of Microgels

A hydrogel in oil emulsification using microfluidics is praised for its production of highly monodisperse microgels in a high-throughput fashion. To recover the microgels for downstream applications, the emulsion must be broken to separate the water and oil phases. Generally, this separation can be achieved using chemical breaking, electrostatic displacement, or washing. All three can be performed either on-chip or in bulk after droplet collection. For off-chip chemical breaking, destabilizing of droplet surface tension using 1H,1H,2H,2H-perfluoro-1-octanol (PFO) is very common practice for fluorinated oils (Karbaschi et al., 2017). This chemical displaces the surfactants in the oil forcing the droplets to coalesce, with the advantages of being quick and easy. As a downside, the approach is generally regarded to affect viability of vulnerable cells. Therefore, a chemical-free alternative can be washing (Chokkalingam et al., 2013) or the use of electrostatics to be gentler on cells (Chokkalingam et al., 2014). On-chip recovery of microgels can also be achieved using PFO (Shao et al., 2020) and electrostatic manipulation to allow direct recovery of microgels in water. The electrostatic approach can either be used to force droplets to coalesce (Huang et al., 2015) or the charge difference can be used to push/pull hydrogels across an oil–water interface (Huang and He, 2014). The latter technique has recently been demonstrated by White et al. as an automated method to selectively sort cell-containing microgels from empty microgels (Wang et al., 2021), thus increasing downstream efficiency and avoiding empty droplets. For washing, on-chip designs have been proposed to get microgels from the oil phase into the water phase. Passive methods use the interfacial tension of the water–oil droplets to allow microgels to merge into a parallel flowing extraction aqueous phase (Wong et al., 2009; Deng et al., 2011). More active approaches use filtering (Bavli et al., 2021) or slow infusion of the aqueous phase (Tang et al., 2009).

## Hydrogel Droplet Single-Cell Applications and Analysis

The interest in single-cell analysis has spiked over the past years because of highly heterogenous cell populations being discovered and proving their importance (Altschuler and Wu, 2010; Satija and Shalek, 2014). Droplet microfluidics allow for a high-throughput approach for single-cell encapsulation and thus isolation, creating reproductive nano-bioreactors facilitating, for example, single-cell sequencing. Furthermore, incorporation of hydrogels allowed control over mechanical properties at single-cell resolution.

### Single-Cell Sequencing

**FIGURE 3:**
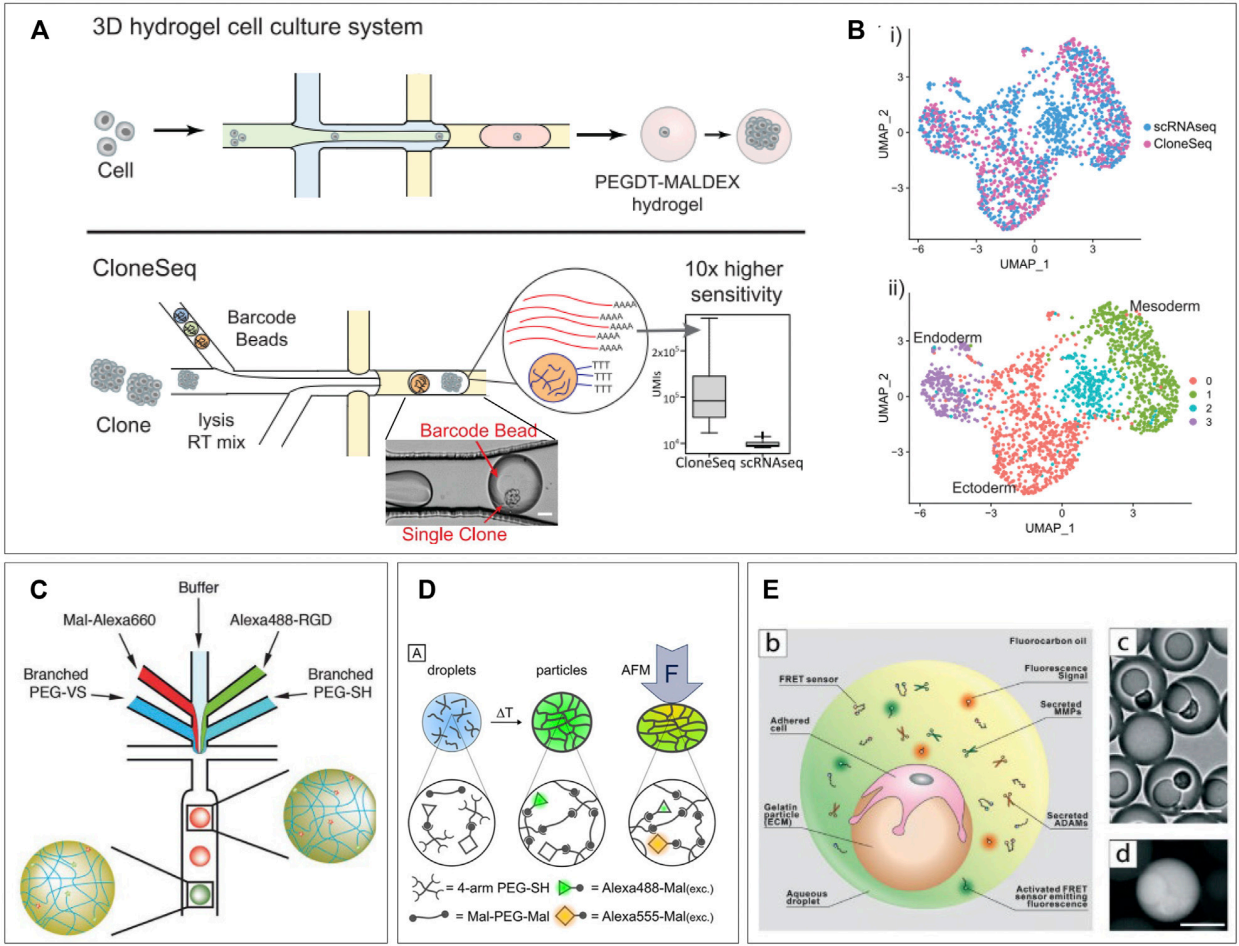
Single-cell applications. **(A)** CloneSeq platform: single-cells are encapsulated in a hydrogel droplet and cultured to form a population of clones. This population is sequenced using an adapted sequencing protocol. **(B)** CloneSeq protocol exhibits improved separation of different stem cell differentiation states compared to conventional single-cell RNA sequencing (Bavli et al., 2021). **(C)** Microfluidic device for production of a library of droplets with unique mechanical and functionalization properties, which can be detected based on fluorescent signature. Adjusting the ratios of branched PEGs and the fluorophore channels allows tuning of droplet properties during production (Allazetta et al., 2017). **(D)** PEG-based hydrogel droplets incorporated with FRET pairs display fluorescence as a result of deformation, creating the potential to measure cell-exerted forces in-droplet (Neubauer et al., 2019). **(E)** Hydrogel droplets with attached cells are re-encapsulated in droplets along with FRET sensors which become fluorescent when bound by various proteases. This platform allows the single-cell measurement of proteases on different types of tumor cells to probe their metastatic behavior (Wang et al., 2021).

Being able to discern which RNA molecules originate from which cell, while maintaining high throughput, has likely been the biggest challenge in performing single-cell RNA (scRNA) sequencing. Lysis of cell suspensions will result in an indiscernible mixture of nucleic acids. Therefore, the first published case of scRNA sequencing performed lysis of just 1 cell within just one tube (Islam et al., 2011). To increase throughput, this was followed up by parallel processing in well plates (Klein et al., 2015). Labeling of RNA strands in wells allowed for bulk polymerase chain reaction (PCR) and retracing cell identity after sequencing. However, this approach was still physically limited by wells’ plate size. In 2015, two separate publications described the application of microfluidics droplet encapsulation to process single-cells with high througput (Leng et al., 2010; Macosko et al., 2015). Co-encapsulation of cells and functional beads allowed for cell lysis, labeling of strands, and reverse transcription inside the droplets, before amplifying the transcripts with PCR in bulk. These applications used droplets for labeling purposes only and performed PCR afterward. To perform PCR in droplets, agarose droplets have been proposed to entrap large molecules of nucleic acid. Such techniques were reported for applications like single-cell molecule amplification (Novak et al., 2011), single-cell DNA trapping (Zhu et al., 2012), or rare pathogen detection (Zilionis et al., 2016). A more recent application of RNA sequencing using hydrogel presented “CloneSeq”, which utilizes in-droplet labeling but with hydrogel droplet pre-cultured single-cell clones to improve sequencing sensitivity (Zhao et al., 2021). Bavli et al. performed a first round of single-cell encapsulation of barcoded cells in PEGDT-Maldex hydrogel droplets, followed by a period of expansion to obtain a clump of clone cells from a single mother cell. The clone-cell clump is then re-encapsulated along with a barcoded bead as in the InDrops protocol (Kumachev et al., 2011) (Figure 3A). Their approach exploits the fact that clonal daughter cells maintain transcriptional similarities, to obtain a 10-fold larger library of unique RNA transcripts allowing for an increased sensitivity. This allowed them to prove that 3D culturing in these microgels maintains more cellular stemness compared to bulk 2D culturing. In addition, they showed that in a mouse model of embryonic stem cells, CloneSeq was much more capable of discerning endo-, ecto-, and mesoderm differentiation states compared to conventional scRNA sequencing (Figure 3B). Also, in the detected differentiation states much more significantly enriched early differentiation genes were measured. In this approach, the implementation of hydrogel facilitates the expansion and spatial control of single-cell clones while allowing re-encapsulation with barcoded beads for sequencing with improved sensitivity. It underlines the potential for hydrogel droplets to improve similar novel techniques while providing suitable culture conditions for cells.

### Tuning Hydrogel Mechanical Properties for Screening of Cellular Responses

Incorporation of hydrogels in microfluidic droplets has allowed for control over an extra parameter: mechanical properties. Tuning of hydrogel stiffness and elasticity is achieved by varying polymeric components and has been previously performed in droplets (Rahimian et al., 2019; Dagogo-Jack and Shaw, 2017). This allows for several directions of analytic tools, from testing individual cell forces (Allazetta et al., 2017), to screening gradients of hydrogel stiffness (Zhao et al., 2016), and the resulting cell responses (Nan et al., 2019). Apart from creating separate batches of hydrogels with varying properties, within a batch gradients of hydrogel stiffness can be screened. By including an extra dispersed phase inlet for different polymeric components and adjusting ratios, hydrogels can be obtained within a wide spectrum of mechanical properties. Kumachev et al. demonstrated this technique in 2011 by combining two streams of different agarose concentrations to create microgels with varying stiffness, suitable for cell encapsulation (Dagogo-Jack and Shaw, 2017). Allazetta et al. adapted the approach by using separate inlets for 4-armed PEG macromers and 8-armed PEG macromers plus a third inlet for red fluorescent maleimide groups (Rahimian et al., 2019). By changing the flow speed ratio between the PEG channels and the fluorescent channel, hydrogels with varying Young’s moduli and reversely correlated fluorescent intensity could be created. These values were shown to correlate linearly allowing for a direct readout of the stiffnesses per individual microgel. Furthermore, by adding two extra dispersed phase inlets, one for buffer and one for green-fluorescent RGD molecules, microgels with tunable and detectable amounts of RGD, covalently build into the polymer structure, could be produced. In a final more complex microfluidic design, this allowed for independent programming of microgel stiffness and RGD-bioactivity along with direct fluorescent readout of exact microgel properties (Figure 3C). In addition, they demonstrated the potency of their platform by producing 100 unique microgel compositions with different functionalization and stiffness. Such tunable libraries of droplets with unique mechanical and chemical characteristics are a potential asset for screening of cell responses to different materials. Other recent research shows potential for in-droplet measurement of such cellular behavior. Neubauer et al. developed an interesting approach to a construct in which direct measurement of exerted forces by single cells can be possible (Allazetta et al., 2017). Their PEG-based hydrogel droplets were incorporated with Alexa488 and Alexa555 as Förster resonance energy transfer (FRET) pairs. These molecules will undergo a fluorescence shift when they are moved into closer proximity. Therefore, deformation of the hydrogel droplets will results in a fluorescent readout (Figure 3D). Although only tested with atomic force microscopy indention, the technique holds promise for real-time visualization of cell forces as the polymers are easily functionalized with cell-binding sites. A recent work by Wang et al. also utilizes FRET signals to detect single-cell responses to differences in mechanical properties of droplets. They produced gelatin beads onto which single cells were allowed to adhere. After adherence, the beads are re-encapsulated in microfluidic droplets along with FRET sensors. These sensors consist of a FRET pair which can be cleaved by specific cell-secreted proteases causing a fluorescent shift. They report on the effects ECM stiffness has on single tumor cells and their production of metastasis-related proteases (Nan et al., 2019). They created gelatin microgels with tunable stiffness onto which three types of human breast cells could adhere: non-tumorigenic cells, non-metastatic cancer cells, and metastatic cancer cells. By re-encapsulating these along with the FRET sensors, the single-cell expression of MMP2, MMP3, MMP9, and ADAM8 could be measured based on droplet fluorescence (Figure 3E). This gave insight into heterogenous cell responses to substrate stiffness correlating to metastatic or non-metastatic behavior. Such research is of high value as therapy resistance is generally attributed to tumor heterogeneity (Meacham and Morrison, 2013; Xi et al., 2017).

## Hydrogel Droplets for Cell Pairing Applications and Analysis

Just like with trap- or well-based microfluidics, droplet-based microfluidics can be utilized to pair cells in close confinement. This relatively untapped category of applications is specifically promising in combination with hydrogels as it allows permanent spatial control, independent of emulsion integrity. This enables delivery of cell nutrients, allows for downstream processing, or co-analysis, all at a high throughput.

### Co-Encapsulation Efficiency and Deterministic Encapsulation

**FIGURE 4:**
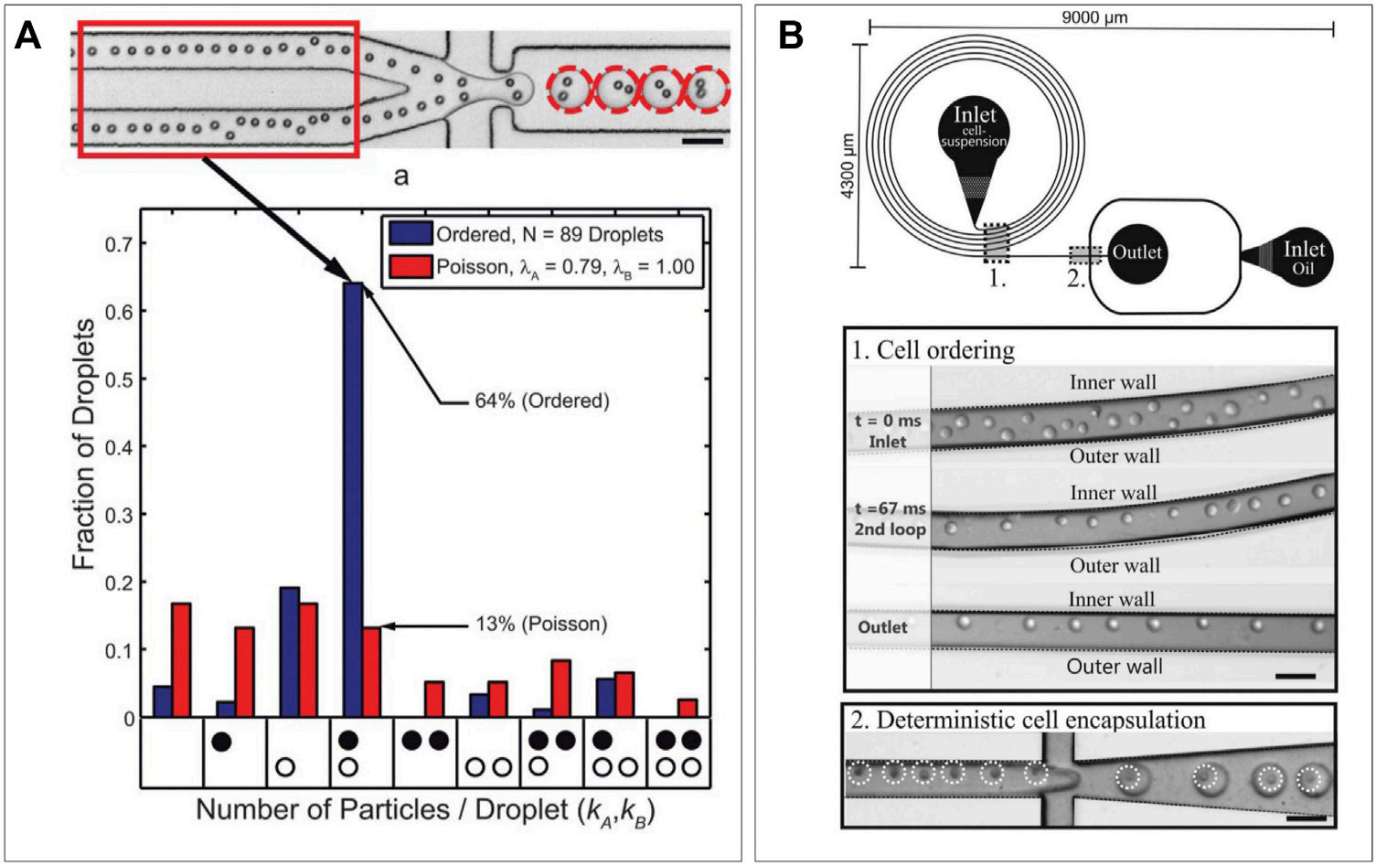
Deterministic encapsulation. **(A)** Radial displacement leading to a particle train which can be synced with frequency of production rate to obtain increased cell-pairing efficiency (Lagus et al., 2013). **(B)** Deans flow in spiral shaped channels can form particle trains on the inner wall, which can be used to obtain deterministic encapsulation (Kemna et al., 2012).

When co-encapsulating a pair of heterotypic particles, the Poisson distribution proves a difficult hurdle (Collins et al., 2015), forcing a trade-off between either too few heterotypic pairs or too many droplets containing homotypic particles. In random encapsulation with a mixture of particles only a small fraction of droplets will contain exactly one particle of each type, which can only be optimized to a certain extent based on concentration and droplet size. Droplets containing the desired number of particles can be sorted on-chip (Hu et al., 2015; Segre and Silberg, 1961), but this will only improve purity and not increase production rate. Therefore, advances have been made in deterministic encapsulation of particles in droplets to skew the Poisson distribution into a desired direction. To achieve this, spatial control of particles inside microfluidic channels is required, which can be achieved *via* inertial focusing through radial particle displacement in long channels (Dean, 2009) (Figure 4A), or Deans flow in spiral-shaped channels (Russom et al., 2009; Kemna et al., 2012) (Figure 4B). Both will result in ordering in an equally spaced train of particles, and when spacing is matched with frequency of droplet production, this can double the efficiency of single particle encapsulation compared to random Poisson encapsulation (Lagus and Edd, 2012; Lagus and Edd, 2013). When ordering is performed on particles in two different inlets, the encapsulation efficiency of two unique particles can be increased to around five times the efficiency of random Poisson encapsulation (Yaghoobi et al., 20202020; Duchamp et al., 2021). Performing such inertial ordering requires relatively simple device designs while maintaining the high-throughput nature characteristic to droplet microfluidics. Other more complex approaches utilize droplet merging to obtain cell pairs more efficiently. These can be performed either using traps (Schoeman et al., 2014; Chung et al., 2017) or by merging droplets inside the device channels (Wimmers et al., 2018). However, results from these efforts have been limited by on-chip capacity or efficiency and, thus, fall short compared to deterministic encapsulation.

### Pairing for Single-Cell Immune Assays

#### Cell–Cell Pairing

**FIGURE 5:**
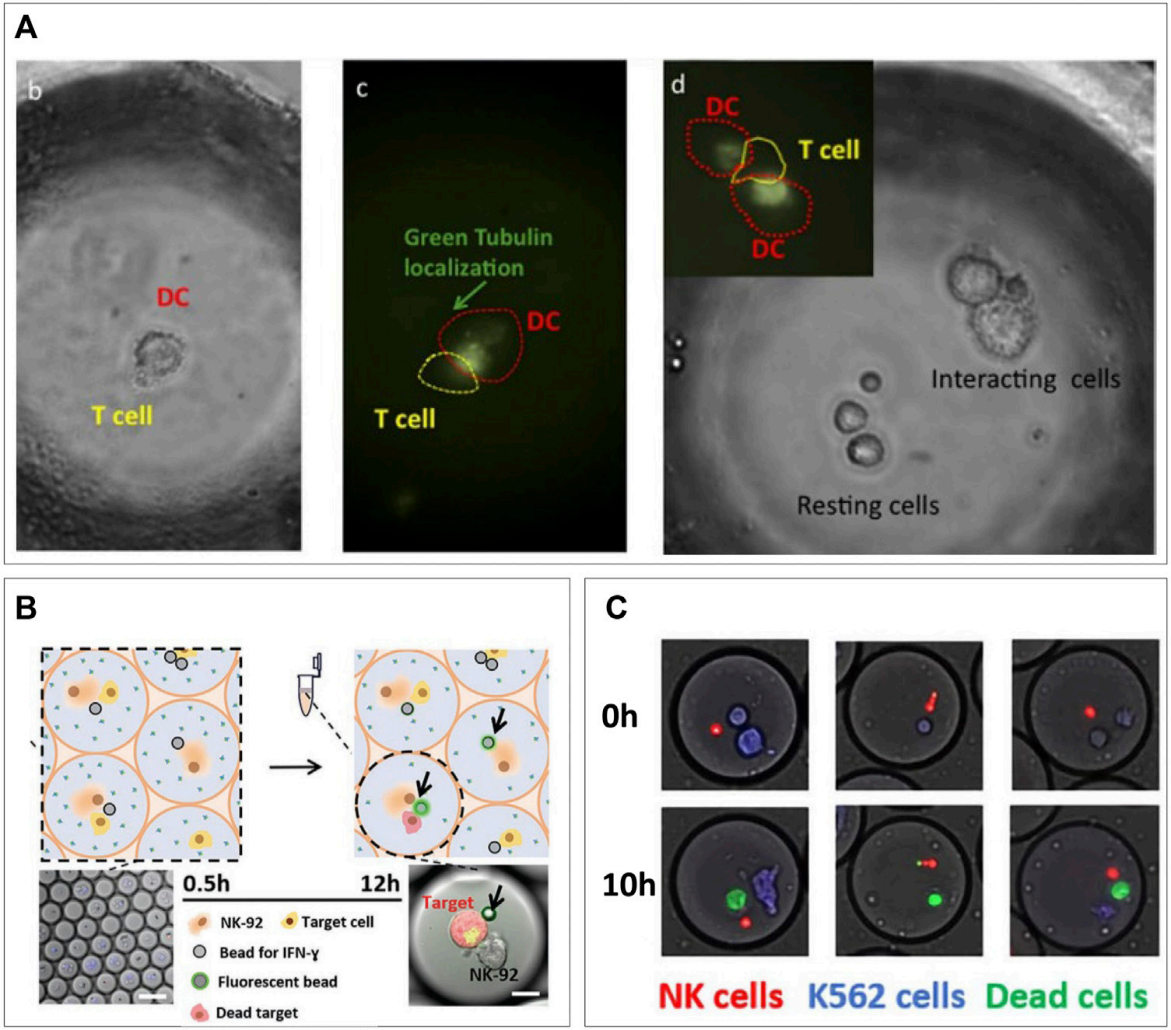
Cell–cell pairing for single-cell immune interactions. **(A)** Microscopic visualization of DC/T-cell interactions. Tubulin localization indicates presence of immune synapses present at different ratios of cells (Konry et al., 2013). **(B)** Monitoring of IFN-γ secretion during NK/tumor cells killing interaction (Antona et al., 2020). **(C)** Primary NK cells killing tumor cells in droplets (Subedi et al., 2021).

The ability of microfluidic droplets to bring two heterotypic cells in confined proximity finds an ideal application in fundamental immunological research. Many immune responses are heavily dependent on cells being adjacent for paracrine signaling or even connecting for juxtacrine signaling. High-throughput droplet encapsulation was readily proven an ideal method to screen immune cell heterogeneity in response to paracrine ques, as modeled by co-encapsulation of various stimuli (Tiemeijer et al., 2021; Konry et al., 2011; van Eyndhoven et al., 2021; Konry et al., 2013). Co-encapsulation allows similar setups for investigating contact-dependent juxtacrine interactions referred to as immune synapses. These have been readily investigated at single-cell level in context of contact-dependent activation (Sarkar et al., 2015; Antona et al., 2020b), and contact-dependent cytotoxicity (Subedi et al., 2021; Sarkar et al., 2017; Dik et al., 2005). The first of the two arises primarily between antigen-presenting cells and lymphoid cells to initiate the adaptive immune response. When dendritic cells (DCs) use the immune synapse to present T cells with antigens, T cells are activated, start proliferating, and become potent killers of virus-infected cells or tumor cells. Not only due to the immense T-cell receptor variation (stPaul and Ohashi, 2020) but also because of the high degree of T-cell heterogeneity (Woodland and Dutton, 2003b; Dura et al., 2015; Ma et al., 2021), this can be a very heterogenous process, in which droplet-based single-cell approaches could improve fundamental research greatly and pave the way for high-throughput screening for therapeutic applications (Hondowicz et al., 2012; Shahi et al., 2017; Sarkar et al., 2016). Konry et al. co-encapsulated mouse DCs and T cells and visualized them in droplets, where they could observe formation of immune synapses based on tubulin localization (Sarkar et al., 2015) (Figure 5A), demonstrating that these cellular interactions can be monitored real-time using microscopy in droplet confinement. Later, in-droplet research included detection of synapse-based activation *via* dynamic calcium signals (Antona et al., 2020b) and demonstrated that synapse duration is dependent on antigen presence (Vanherberghen et al., 2013). In addition, they showed contact-dependent T cell–mediated tumor cell cytotoxicity in droplets, which they were able to boost or inhibit *via* added stimuli or cytokine inhibition, respectively (Vanherberghen et al., 2013). In addition to T cells, natural killer (NK) cells are very potent cytotoxic cells, which on contact with a target cell exhibit antigen-independent killing demonstrate serial killing and have been shown to behave with a highly heterogenous nature (Guldevall et al., 2016; Antona et al., 2020b). The immune synapses *via* which their cytotoxicity is elicited are varying in duration and can occur several times for a single target cell or several target cells resulting in serial killing. These repeated killing synapses were studied in droplets between NK cells and different types of tumor cells by Antona et al. (2020a). They encapsulated varying numbers or target cells in droplets of different sizes and showed that serial killing was controlled by both variables. In a different application, they complemented the cytotoxicity assay with monitoring of IFN-γ secretion by NK cells (Sarkar et al., 2020) (Figure 5B), which allowed them to show that only about half of the NK cells killing K562 tumor cells produces IFN-γ in the process, underlining their heterogenous nature. As these assays are all monitored using microscopy over time, manual analysis is tedious and can potentially be biased. Subedi et al. developed an automated real-time analysis script which allows for these killing assays to be performed in an unbiased manner (Sarkar et al., 2017). In addition, they showed the suitability for this platform for killing by primary isolated human NK cells (Figure 5C). These droplet techniques for analysis of immune synapses have proven valuable in progressing of basic knowledge of immune cell interaction. They have underlined its potential for elucidating heterogeneity and proven their usefulness in finding sub-populations of therapeutic interest. Nevertheless, in terms of the high-throughput potential droplet-microfluidics natively has, there is still much progress to be made. These techniques rely on microscopic visualization in observation wells and chambers for the analysis of pairs which allows measurement of several hundred (Scanlon et al., 2014; Antona et al., 2020a) to few thousand events (Sarkar et al., 2017), respectively, but are thereby inherently limited in its throughput to the size of the microfluidic device. This is where application of hydrogel in droplets can allow for upscaling to achieve not only very high throughput but also recovery of droplets from the experimental setup and maintaining the spatial bond between cell pairs after de-emulsification of droplets. This has been previously performed in screening of bacterial colonies. Scanlon et al. co-encapsulated antibiotic expressing *E. coli* in agarose droplets along with *S. aureus* and a fluorescent viability marker (Kitaeva et al., 2020). The combination of production rates up to 3,000 drops/s and off-chip collection in centrifuge tubes allowed theoretical unlimited flow through. An analysis was performed by gelation of the agarose droplet, de-emulsification, and whole droplets measurement of *S. aureus* viability using flow cytometry. This ultrahigh-throughput allowed for the detection and sorting of up to five million clones/day to screen for potential novel antibiotics. Such upscaling is expected to greatly benefit the field of immune therapy by allowing ultrahigh-throughput of cell pairs and their immune synapses, which will improve drug testing (Jiang et al., 2019b) and help in the discovery of tumor neoantigens (Heo et al., 2020) and specialized cell populations (Altschuler and Wu, 2010).

#### Cell–Reporter Pairing

**FIGURE 6:**
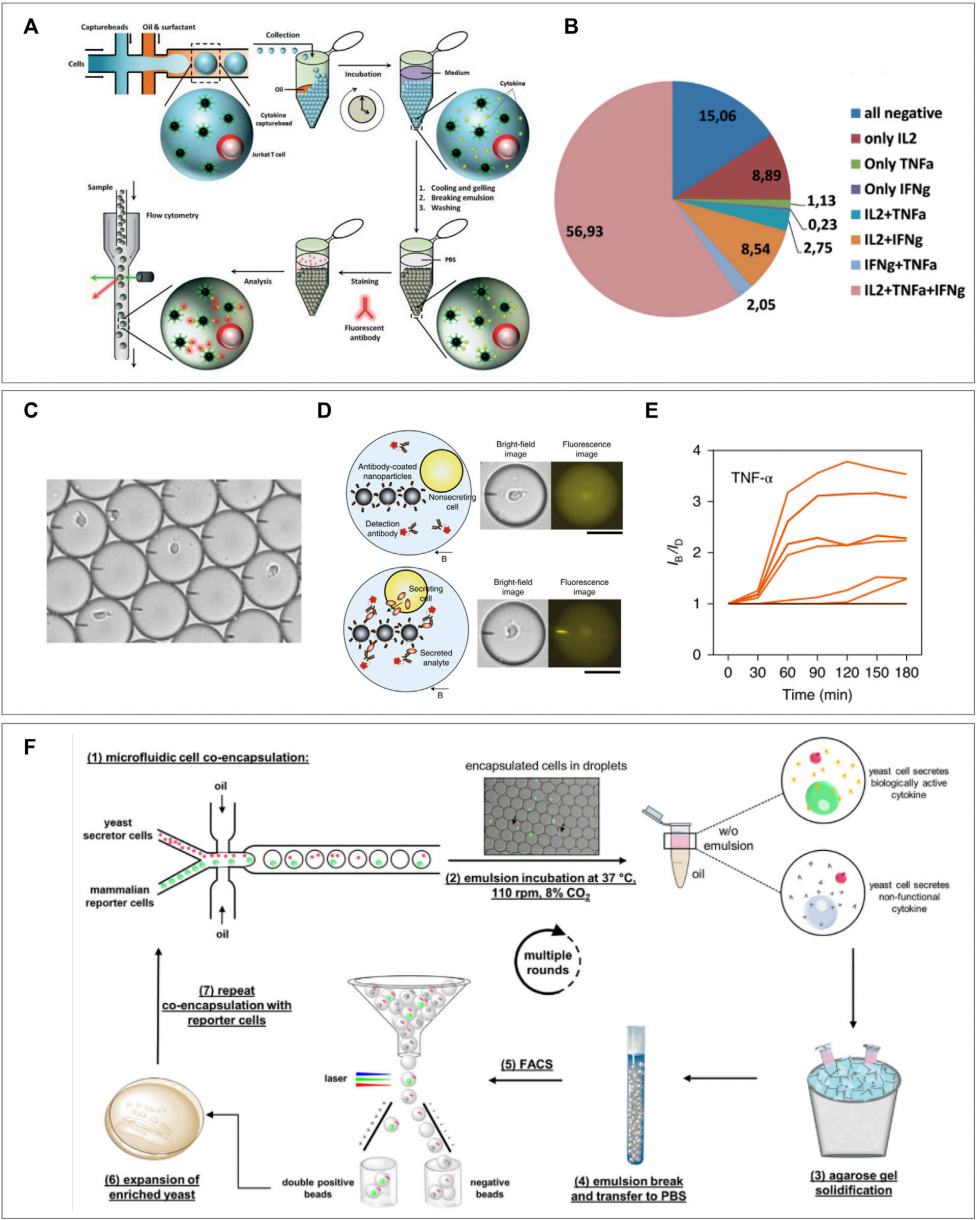
Cell-reporter pairing for immune assays. **(A)** Capture beads specific for three different cytokines are co-encapsulated in hydrogel droplets, after gelation and emulsion breaking the whole droplet can be stained for captured cytokines and measured using flow cytometry. **(B)** Secretional heterogeneity of Jurkat T cells can be detected using this method of multiparameter single-cell cytokine detection (Chokkalingam et al., 2013). **(C)** Bead-line encapsulation evades the Poisson distribution as every droplet will contain around 1,000 nm-sized capture beads. **(D)** Cellular cytokine secretion can be observed and **(E)** quantified over time by measuring translocation of co-encapsulated fluorescent antibodies (Bounab et al., 2020). **(F)** Yeast cells are co-encapsulated with murine IL-3 reporter cells. Gelation of droplets and de-emulsification allows flow cytometric sorting of IL-3 producing yeast cells. Expansion and repeating of procedure allows enrichment of secreting yeast cells (Yanakieva et al., 2020).

In addition to using pairing of heterotypic cells in droplets to measure their interactions, a cell of interest can also be encapsulated alongside a “reporter” particle or cell. After periods of culture/stimulation fluorescent signal can be measured on the reporter cell either due to translocation of co-encapsulated markers (Fang et al., 2017; Sarkar et al., 2020; Bounab et al., 2020; Gérard et al., 2020; Garti and Bisperink, 1998) or by direct staining after de-emulsification (Yanakieva et al., 2020; Chokkalingam et al., 2013). The main advantage of co-encapsulation of fluorescent markers is that microscopic imaging allows continuous measurement to obtain temporal information. For staining after de-emulsification, a hydrogel is indispensable as it maintains the spatial coupling of the interrogated cell and reporter particle. This latter approach was demonstrated in research by Chokkalingam et al. where Jurkat T cells were co-encapsulated with functionalized cytokine capture beads (Chokkalingam et al., 2013) (Figure 6A). Beads contained antibodies specific for IL-2, TNF-α, and IFN-γ so that cytokines secreted by co-encapsulated cells would be captured on the beads during incubation in the droplets. As they co-encapsulated a solution of low melting-point agarose, simple cooling of the droplets after culturing allowed spatial linking of the Jurkat T cells with the capture beads allowing for flow cytometric detection of cytokines for each individual cell. This revealed heterogenous secretion behavior and distinct secretion profiles (Figure 6B). Since analysis for such experiments is performed using flow cytometry, it has the potential to immediately sort cells of interest, as was demonstrated for bacteria by Scanlon et al. Another recently published protocol by Bounab et al. cleverly utilizes fluorescent translocation to monitor timing of cytokine secretion by monocytes and T cells or IgG production by B cells, at a single-cell level (Gérard et al., 2020). In addition, they were able to cheat the Poisson distribution hurdle by co-encapsulating with a high concentration of nanometer-sized magnetic particles. After droplet capture these form a “beadline” in response to a magnetic field, and they will be present in every droplet due to the high concentration of particles (Figure 6C). The magnetic particles are functionalized with cytokine-specific capture antibodies and fluorescent detection antibodies are co-encapsulated in the droplets. Cytokine secretion by encapsulated cells will therefore result in a real-time observable antibody-cytokine sandwich which can be visualized by fluorescent translocation to the beadline (Figures 6D,E). Although the throughput is limited by the size of their observation chamber, the approach still allows for analysis of up to 300,000 single cells per experiment. The versatility of this technique was demonstrated by immobilization of heat killed bacteria on the beadline (Bounab et al., 2020). This “Bactoline” allowed screening of IgG and IgM production by mice B cells before and after immunization, which provided valuable insights into single-cell heterogeneity of antibody specificity, affinity, and cross-reactivity. Apart from beadline measurement in observation chambers, translocation of fluorophores can also be measured in droplets flow on-chip (Garti and Bisperink, 1998). This allowed for direct sorting of B cells producing antibodies to co-encapsulated antigens and sequencing of their IgG sequences. The method was further shown to be suitable for screening of *ex vivo* stimulated human peripheral memory B cells, indicating the potential for these types of techniques to contribute to development of immune techniques.

In addition to reporter particles, several applications have been demonstrated using reporter cells (Garti and Bisperink, 1998; Fang et al., 2017; Yanakieva et al., 2020). Fang et al. used hydrogel droplet sorting to enrich rare antibody clones as produced by transfected yeast cells. They co-encapsulated antibody producing yeast cells with A431 tumor cells expressing the relative antigen. Gelation of the agarose droplets before de-emulsification spatially bound the producer and reporter cell. After de-emulsification pairs of yeast and A431 cells in hydrogel droplet were washed to get rid of non-specific IgG`s and stained using fluorescent anti-human IgG to visualize A431 bound antibodies. During flow cytometric sorting these A431 could be detected based on fluorescence and sorted out. As they were spatially confined to the corresponding yeast cells, these were collected as well, thus increasing purity of yeast cells of interest. More recently, a similar approach using agarose droplets was demonstrated for enrichment of cytokine secreting yeast by Yanakieva et al. (2020) (Figure 6D) Here, however, no additional fluorescent staining was required as they used mCherry positive murine IL-3 secreting yeast cells in combination with murine reporter cells, which expressed green fluorescent protein (GFP) upon stimulation with IL-3. Co-encapsulation of both cells would therefore result in a double positive signal for mCherry and GFP. By flow cytometric sorting of double positive droplets, IL-3 producers could be enriched and expanded before additional rounds of encapsulation with reporter cells. This allowed them to enrich yeast that produced functional IL-3 from yeast producing inactive IL-3 from a 1:10,000 mixture by 70-fold with only two rounds of sorting. This technique of repeated enrichment combined with unlimited throughput proves suitable for efficient selection of yeast clones secreting biorelevant proteins.

## Semi-Permeable Hydrogel Shells

**FIGURE 7:**
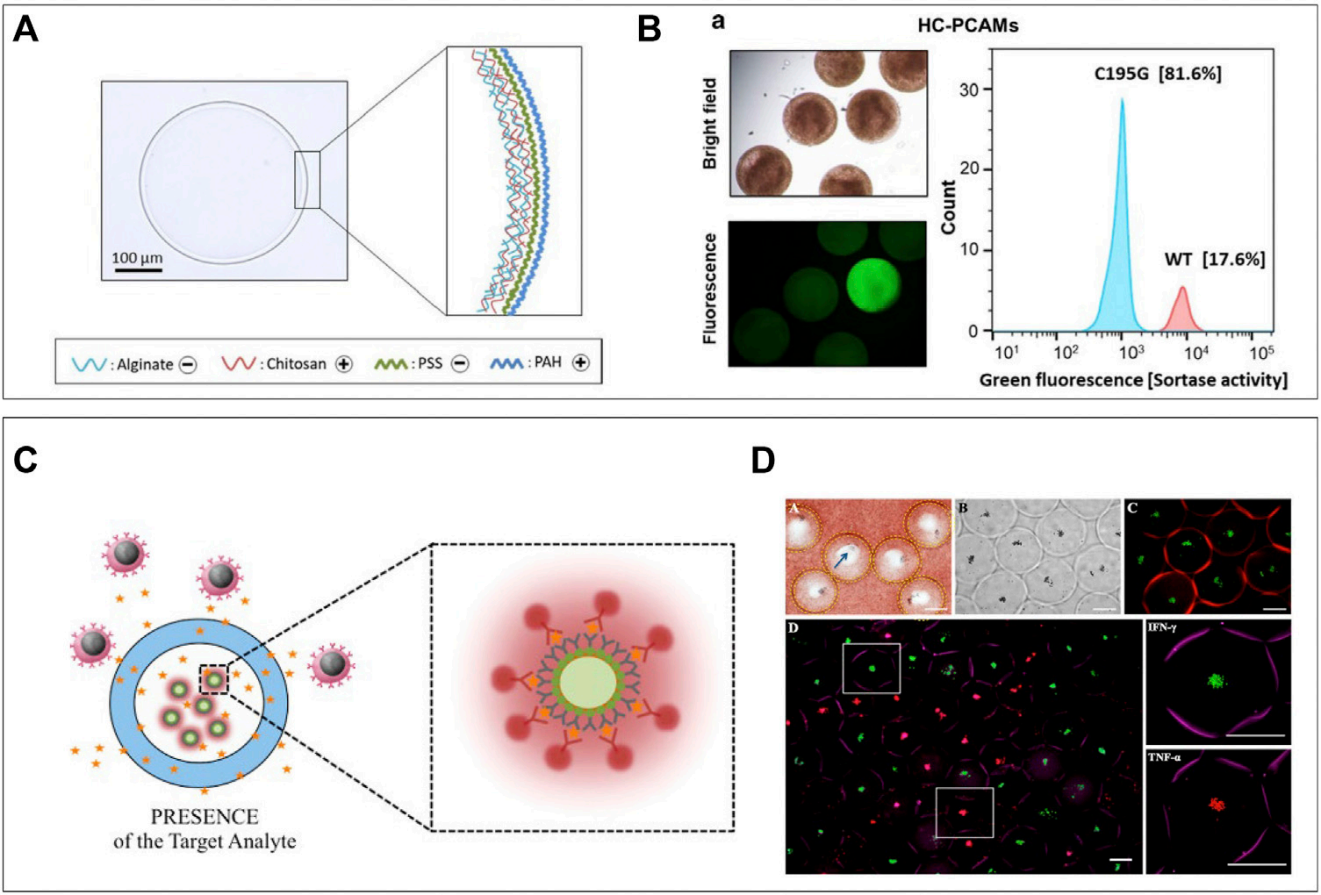
Semi-permeable hydrogel shells. **(A)** Hydrogel shell droplets are coated with polyelectrolytes to decrease permeability. This successfully allows the diffusion of nutrients and lysis buffer, but keeps DNA inside after lysis. **(B)** Particles can be analyzed and sorted based on fluorescence in a large particle sorter (di Girolamo et al., 2020). **(C)** Selectively permeable hydrogel shell particles used as sensors preventing cells to reach the microbeads but letting cytokine and detection antibodies diffuse in. **(D)** Sensor particles submerged in whole blood and after washing and incubation with detection antibodies. IFN-γ specific particles are green, and TNF-α specific particles are red (Rahimian et al., 2019).

The production of double emulsions of water and oil has been investigated for a few decades (Chong et al., 2015), but the rise of droplet microfluidics has enabled stable and high-throughput production (Huang et al., 2017). Adding hydrogel into the “shell” has resulted in the facile production of semi-permeable particles suitable for cell analytical procedures (Choi et al., 2016). Shells can be obtained by direct on-chip formation of hydrogel layers (Cai et al., 2019; Mak et al., 2008), deposition of silica (Mazutis et al., 2013), or polyelectrolytes (Fischlechner et al., 2014; Ferreira et al., 2013) after droplet production. The latter is used to drastically decrease permeability to selectively retain macromolecules but allow diffusion of small molecules. di Girolamo et al. (2020) utilized the technique to further increase the selectivity of their chitosan-shell alginate-core droplets, which allowed for retention of ∼30 kDa molecules and diffusion of ∼1 kDa molecules. First, they produced alginate beads using common microfluidic techniques that allowed for cell encapsulation. These microgels could then be coated with chitosan, after which the alginate core was dissolved using sodium citrate. These hollow core chitosan–alginate capsules could then be coated with subsequent layers of poly(styrene sulfonate) and poly(allylamine hydro-chloride) to control permeability (Figure 7A). Their particles were suitable for encapsulation and cultivation of *E. Coli* and by culturing these from single-cells to over two million per capsule, they demonstrated the shell did not prevent diffusion of nutrients and oxygen. Subsequently lysis buffer could be washed in to digest the cells, after which cellular large proteins and DNA were retained by the coating. Fluorescent probes specific for enzymes of interest could be washed in to detect which droplets contained cells producing these enzymes. Such an approach can be combined with fluorescence-based sorting techniques to facilitate directed evolution (Figure 7B). Hydrogel shells without polyelectrolyte generally have larger pore size and are thus selective to a different range of molecular sizes. A dextran-core PEGDA-shell particle was designed by Leonaviciene et al. which they approximated to have a pore size of around 30 nm, thus successfully retaining DNA fragments of around 340 kDa and higher (Leonaviciene et al., 2020). This allowed them to wash lysis buffer and genome amplification reagents in and out of the particles in a multistep process. As the amount of reagent was not dependent on the droplet volume, this resulted in a 2-fold higher yield of DNA macromolecules compared to conventional water-in-oil droplets, in which supply of reagent is limited by what is initially encapsulated. By using their approach to cultivate biodegradable plastic producing bacteria, they demonstrated detection of functional products as a readout, in addition to amplification of genomic contents. An opposite approach of such semi-permeable shells is to prohibit cells from entering the core but allowing specific analytes in. Rahimian et al. applied this concept in an immunoassay for the *ex vivo* analysis of whole blood for specific secreted factors (Zhu, 2010). A thin PEG shell was produced surrounding a 400-μm size particle containing antibody-coated beads specific for both TNFa and IFNy, which are important pro-inflammatory markers. The coating prevented leukocyte interactions with the detection beads while secreted cytokines could enter and bind the beads (Figure 7C). Retrieval of beads by simple filtering, followed by addition of fluorescent detection antibodies allowed for cytokine analysis without preceding cell-separation steps. They demonstrated the feasibility of the platform by detection of IFNy in blood of patients with a latent tuberculosis infection (Figure 7D). Comparison to the current gold standard method resulted in 11 out of 14 patients getting the correct diagnosis, and the three incorrect diagnoses were false-negatives. Although most of the aforementioned methods are executed with bacteria, these can easily be translated to mammalian cells. Such approaches would yield unique opportunities for prolonged single-cell cultures or single-cell cloning due to unlimited inflow of cell nutrients. In addition, hydrogel-shell particles can be utilized as miniaturized bioreactors for chemical reactions such as PCR.

## Hydrogel Droplets for Cocultures

Although exact cell pairing efficiency remains a challenging hurdle, various efforts have been made to utilize hydrogel droplets for cellular coculture with multiple cells. As cellular interactions are fundamental in biology, downscaling can help understand them at the most basic level. Hydrogel droplets facilitate this type of research by bringing few-cells in tight proximity and keeping them there after de-emulsification. As this allows inflow of nutrients these cocultures can be maintained over a much longer period (Tumarkin et al., 2011) to study cell–cell effects (Kmiec, 2001). A great example is the effect of cocultures of liver cells on activity and viability of hepatocytes, as such interactions have proven complex (Kmiec, 2001) and hepatocytes alone display very little liver specific functions (van Poll et al., 2006; Cho et al., 2010). These cocultures were demonstrated in organoids previously (Darakhshan et al., 2020), but for further downscaling and increased throughput, droplet microfluidics was applied. Cocultures of hepatocytes were produced as spheroids (Chan et al., 2016) or in a core-shell orientation (Lu et al., 2015; Chen et al., 2016). Chan et al. used a coculture of endothelial progenitor cells and hepatocytes in hydrogel droplets with a mix of alginate and collagen type I^172^. They utilized alginate-in-oil-in-water double emulsions to produce droplets and eliminated the oil phase in the presence of calcium ions to gelate the droplets. They showed that even at these miniature scales of only several cells, coculture effect was observed in increased production of several functional liver metabolites. Furthermore, tuning composition of alginate and collagen hydrogel in droplets proved to outperform the golden standard for 2D hepatocyte culture in terms of metabolite production (Dunn et al., 1991; Chan et al., 2016). In research by Chen et al., it was shown that in shell-core hydrogel droplets coculture different types of cells could be confined to either encapsulation in the core or shell part of the droplets (Chen et al., 2016). They were able to achieve this distribution by quick on-chip gelation of the alginate shell by acidification of calcium–EDTA complexes using a 4-inlet microfluidic device. In addition, they showed that cell viability was unaffected, and that coculture resulted in increased secretion of liver specific metabolites albumin and urea. Such miniaturized *in vitro* coculture models prove a valuable tool for understanding basic biology, high-throughput drug screening (Darakhshan et al., 2020) or potentially therapeutic applications.

## Concluding Remarks

Single-cell technologies are increasingly more integrated into modern cell biological research. As this leads to discovery of more heterogeneity and rare subpopulations the need for robust high-throughput platforms becomes clearer. The role of microfluidics in achieving single-cell resolution has been cut out for almost 2 decades due to its possibilities to physically manipulate single cells using fluids. Droplet-based microfluidics has obtained its own prominent seat within this field due to the high-throughput and reproducible nature of the resulting micro-scale emulsions. An impressive resume of single-cell applications (Klein et al., 2015; Macosko et al., 2015; Wimmers et al., 2018; Antona et al., 2020b; Bounab et al., 2020; Gérard et al., 2020; Subedi et al., 2021; van Eyndhoven et al., 2021) predicts that droplet encapsulation will continue to play an important role in isolation of individual cells over years to come. A promising addition to the field is the application of hydrogel to produce microgels for single-cell encapsulation. This allows the development of various novel techniques, has the potential to greatly increase throughput, provides improved spatial control, and gives encapsulated cells a mechanically active environment.

Encapsulation of cells in aqueous droplets will result in cells being in forced suspension. This is fine for non-adherent cells but might affect the viability and phenotype of adherent cells that require mechanical stimuli and attachment molecules (Gilmore, 2005; Taddei et al., 2012; Tiemeijer et al., 2021). As hydrogels often mimic the stiffness of extracellular matrix and can be functionalized to facilitate adherence, production of microgels can open the technology to a broader range of cell applications. Current research is still based on proof-of-principle experiments to explore possibilities for adherent cells. But future work can include more complex experimental setups including tunable mechanical properties (Allazetta et al., 2017) and screening of resulting cellular responses (Neubauer et al., 2019; Wang et al., 2021). Such experiments can gain invaluable insights in the fundamentals of cell interactions with their environment and their responses to stiffness, which will be especially usefully for development of tissue engineering approaches (Liebschner et al., 2005; Kurazumi et al., 2011; Wissing et al., 2017).

Although still limited by the Poisson distribution, cell pairing in droplets has been applied previously to bring two types of cells in isolated proximity (Hondroulis et al., 2017; Antona et al., 2020b; Gérard et al., 2020; Subedi et al., 2021). In conventional aqueous droplets, this pairing can only be maintained if droplets are kept in emulsion; however, this will limit downstream processing and prohibits diffusion of nutrients and additional reagents. Pairing of cells in microgels will result in maintained spatial control after de-emulsification. Continuing the culture of cells in microgels after emulsion breaking allows for paracrine cell communication between droplets and should, therefore, be used with caution. However, the most promising microgel pairing application will be the downstream analysis of cell pairs. Especially in the field of immunology, this will create many new experimental opportunities, as immune synapses are the key to the onset and progression of immune responses. Performing single-cell pairing experiments at a high-throughput scale is therefore indispensable to obtain fundamental knowledge required for designing immune therapeutic techniques.

In conclusion, the field of single-cell droplet-based microfluidics will greatly benefit from incorporation of various types of hydrogels. The addition of these “solids” in microfluidics will improve control and robustness, thereby paving the way for standardized high-throughput screening techniques and immune assays. Although until now most applications have been proof-of-principles and focused on finding possibilities, once matured, these technologies will be highly compatible with the future of cell biological research and immunotherapy.
